# Advanced phasing techniques in congenital skin diseases

**DOI:** 10.1111/1346-8138.17597

**Published:** 2024-12-26

**Authors:** Ken Natsuga

**Affiliations:** ^1^ Department of Dermatology, Faculty of Medicine and Graduate School of Medicine Hokkaido University Sapporo Japan

**Keywords:** epidermolysis bullosa, long‐read sequencing, nanopore sequencing, revertant mosaicism

## Abstract

Phasing, the process of determining which alleles at different loci on homologous chromosomes belong together on the same chromosome, is crucial in the diagnosis and management of autosomal recessive diseases. Advances in long‐read sequencing technologies have significantly enhanced our ability to accurately determine haplotypes. This review discusses the application of low‐coverage long‐read sequencing, nanopore Cas9‐guided long‐read sequencing, and adaptive sampling in phasing, highlighting their utility in complex clinical scenarios. Through clinical vignettes, we explore the importance of phasing in gene therapy design for recessive dystrophic epidermolysis bullosa and the role of revertant mosaicism in therapeutic epidermal autografts. Despite its promise, phasing with long‐read sequencing faces challenges, including low efficiency in enriching target regions and the inherent error rate of nanopore sequencing. Future developments in long‐read sequencing technologies will be critical in overcoming these limitations and expanding the applicability of phasing across various clinical settings.

## 1 WHAT IS PHASING AND WHEN IS IT NEEDED?

**FIGURE 1:**
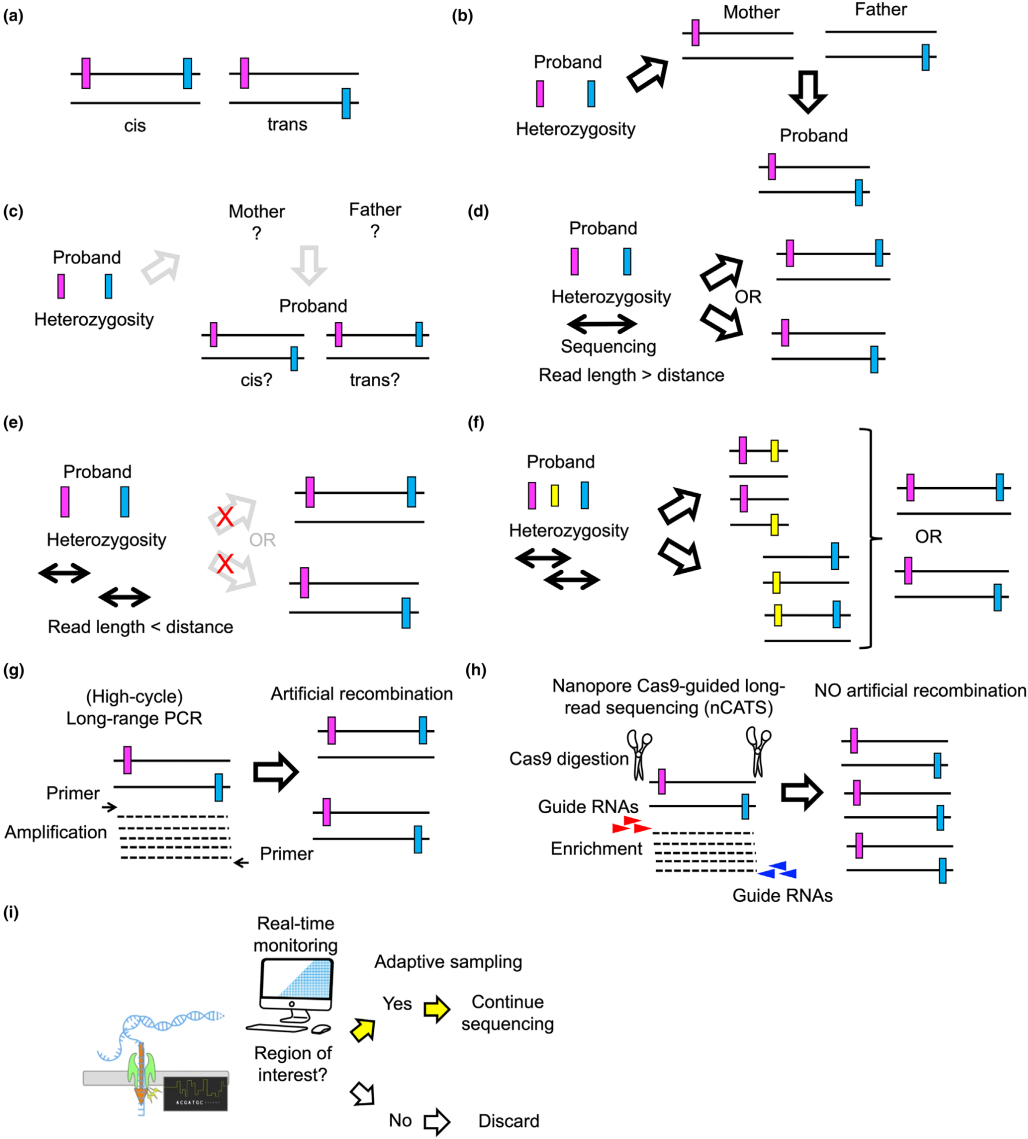
Basics of phasing in autosomal recessive diseases. (a) Cis and trans of two variants, depicted as magenta and blue rectangles. (b) Phasing by inferring through the results of paternal DNA samples. (c) Difficulties in phasing when parental DNA samples are unavailable. (d) Phasing through sequencing when the read length is longer than the distance between the variants. (e) Failure in phasing when the read length is shorter than the distance between the variants. (f) Phasing using another variant (yellow rectangle) by sequencing with a read length that is shorter than the distance between the original two variants (magenta and blue rectangles). (g) Artificial recombination due to a high number of PCR cycles. (h) Nanopore Cas9‐guided long‐read sequencing (nCATS). (i) Nanopore sequencing with adaptive sampling. Nanopore sequencing icon by DBCLS (https://togotv.dbcls.jp/en/pics.html) is licensed under CC‐BY 4.0 Unported (https://creativecommons.org/licenses/by/4.0/).

Phasing is the process of determining which alleles at different loci on homologous chromosomes belong together on the same chromosome, that is, phasing assigns the alleles to their parental chromosome (from the mother or the father). In clinical settings, phasing is important for diagnosing autosomal recessive diseases. These conditions arise from bi‐allelic variants of a disease‐causing gene, with compound heterozygous variants often being more common than homozygous variants, except in cases of consanguinity. When two pathogenic variants are identified within a gene responsible for a disease, it is crucial to determine whether these variants are located on different alleles (in trans) or on the same allele (in cis) (Figure 1a). When the variants are on different alleles, they can indeed cause the autosomal recessive disorder, whereas if they are on the same allele, the variants alone are insufficient to explain the disease phenotype.

Clinicians typically rely on DNA samples from the proband's parents to deduce the proband's haplotype (trio analysis, Figure 1b). However, parental DNA may not always be available, making this phasing approach impractical (Figure 1c). The feasibility of phasing using only the proband's DNA depends on the physical distance between the two variants (Figure 1d,e). Conventional short‐read sequencing technologies (e.g., whole‐exome sequencing [WES], whole‐genome sequencing) are helpful for phasing when the distance between the two variants is within the read length produced by such technologies, which is typically 0.1–0.3 kb (Figure 1d). Even if the distance exceeds the read length, haplotype inference may still be possible through the identification of additional heterozygous variants located between the two primary variants (Figure 1f). However, such instances are uncommon, and accurate phasing often requires long‐read sequencing,^1^ as discussed below.

## 2 WHAT METHODS CAN BE APPLIED TO PHASING?

Recent technological advancements have enabled the acquisition of long‐read sequencing data, such as >10 kb, significantly enhancing our ability to determine haplotypes.^1^ The primary platforms for this purpose are PacBio and nanopore sequencing, provided by Pacific Biosciences and Oxford Nanopore Technologies, respectively.^2^ These platforms differ in their technological approaches: PacBio uses single‐molecule real‐time (SMRT) sequencing, where DNA synthesis is captured by monitoring the fluorescence emitted during nucleotide incorporation by a DNA polymerase. In contrast, nanopore sequencing determines the nucleotide sequence by measuring changes in the electrical current as single DNA molecules pass through a protein nanopore. Each technology has its strengths and limitations, but generally, PacBio sequencing is known for its higher accuracy, while nanopore sequencing offers longer read lengths at a lower cost.

For phasing, low‐coverage long‐read sequencing on genomic DNA (gDNA) is possible, as exemplified in Clinical Vignettes 1 and 2. In contrast, to achieve high coverage, sequencing long‐range PCR products from gDNA is a viable option, provided the length of the PCR products aligns with the genomic distances needed to determine the haplotypes. However, PCR can induce artificial recombination between alleles^3^, ^4^ (Figure 1g), which significantly impacts the accuracy of phasing. To mitigate this issue, the number of PCR cycles can be minimized, although too few cycles may result in insufficient amounts of DNA for sequencing. To address this limitation, nanopore Cas9‐guided long‐read sequencing (nCATS) has been developed, which eliminates the need for PCR.^5^ In this method, gDNA samples undergo a series of processes, including dephosphorylation, Cas9 digestion with guide RNAs targeting regions upstream of the variants of interest, d(A)‐tailing, adaptor ligation, and nanopore sequencing (Figure 1h). This technique allows for the enrichment of regions of interest up to 50 kb, significantly surpassing the amplification capacity of DNA polymerases. Clinical Vignettes 3 and 4 describe examples in which nCATS was applied to phasing in skin diseases.

An alternative method for enriching the genetic region of interest is adaptive sampling (also called selective sampling). This method utilizes the unique capabilities of nanopore sequencing technology to control the movement of individual nucleic acid strands through nanopores, enabling real‐time decisions about which molecules to sequence fully and which to discard (Figure 1i).^6^ Adaptive sampling is implemented through specialized software (e.g., Readuntil^7^). Clinical Vignette 5 shows an example in which adaptive sampling was used in phasing.

## 3 CLINICAL VIGNETTE 1

**FIGURE 2:**
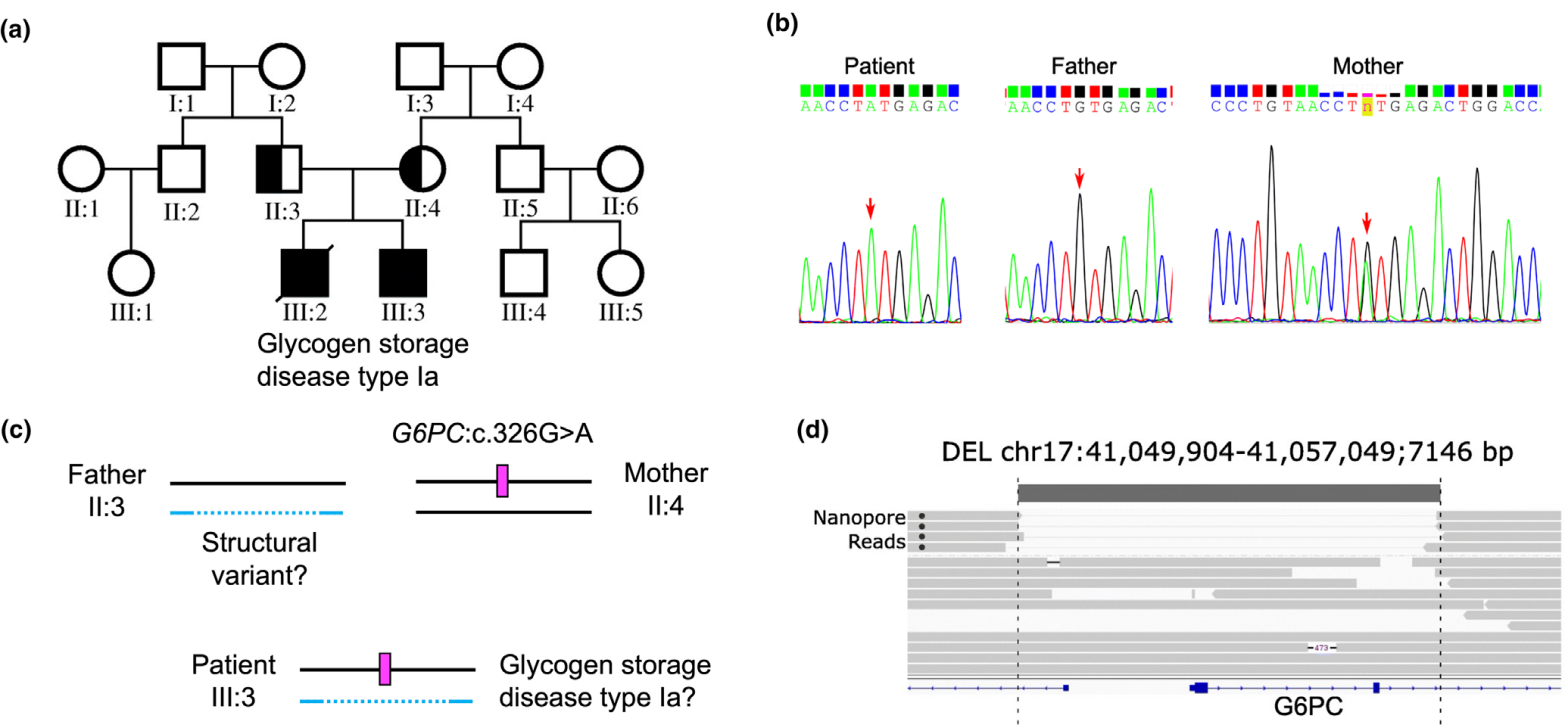
Phasing in glycogen storage disease type Ia using low‐coverage long‐read sequencing. (a) Pedigree of the family with glycogen storage disease type Ia. (b) Sanger sequencing results of the pedigree. (c) Possible diplotypes. (d) Large deletion detected by nanopore sequencing (four reads). Images (a, b, d) reproduced, under CC‐BY 4.0 (https://creativecommons.org/licenses/by/4.0/), from Ref. [9].

Glycogen storage disease type Ia (GSD‐Ia) is a rare congenital disease caused by a deficiency in glucose‐6‐phosphatase.^8^ A previous case study of a GSD‐Ia family (Figure 2a) provided an educational example of phasing.^9^ Although GSD‐Ia is an autosomal recessive disorder requiring biallelic *G6PC* variants, WES and Sanger sequencing of the proband (III:3) identified only a heterozygous variant (c.326G>A) inherited from the mother (II:4). In contrast, the father exhibited a wild‐type *G6PC* sequence (Figure 2b). The authors hypothesized that a structural variant inherited from the father but undetected by WES might account for the development of GSD‐Ia in the proband (Figure 2c). Nanopore sequencing of the proband's gDNA identified a large deletion in the *G6PC* region on the other allele of c.326G>A (Figure 2d). This heterozygous deletion was also present in the father. The detection of the structural variant and simultaneous phasing were possible with low‐coverage reads and proved useful for preimplantation genetic diagnosis in this family. This low‐coverage long‐read sequencing also excluded uniparental isodisomy, a rare condition in which two identical copies of a single homolog of a chromosome from one parent are inherited.^10^, ^11^

## 4 CLINICAL VIGNETTE 2

**FIGURE 3:**
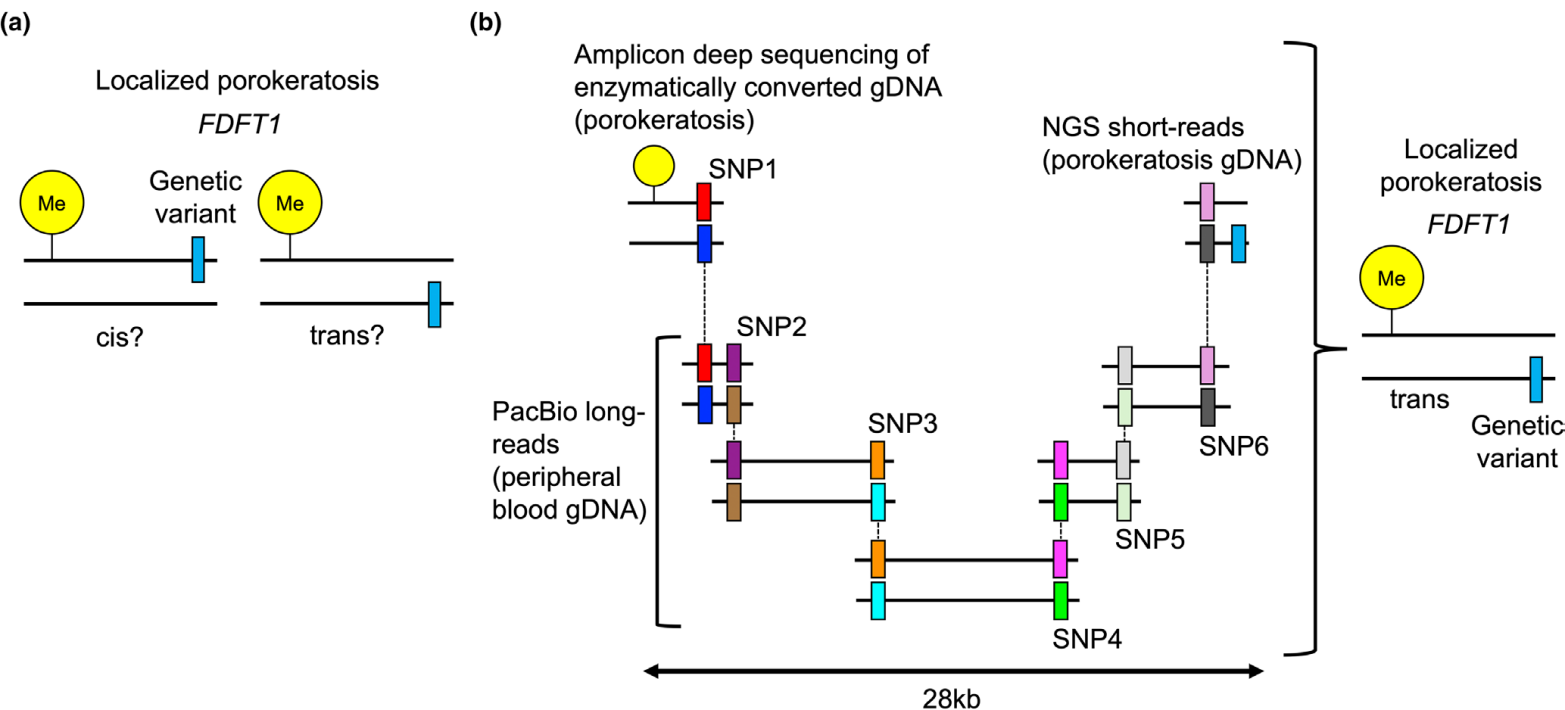
Phasing in localized porokeratosis using a hybrid strategy. (a) Localized porokeratosis with *FDFT1* methylation and genetic variants. (b) Phasing using a combination of amplicon deep sequencing, next‐generation sequencing (NGS), and PacBio low‐coverage long‐read sequencing (2–11 reads). The methylation and genetic variants were present in trans. Images reconstructed from the data on Individual 8, Lesion 4, from Ref. [16].

Porokeratosis is a common hyperkeratotic eruption, which can be disseminated, grouped locally, or solitary. Germline variants in genes involved in the mevalonate pathway (*MVK*, *PMVK*, *MVD*, and *FDPS*) have been reported in porokeratosis patients,^12^, ^13^ with somatic second‐hit variants being responsible for each lesion.^14^, ^15^ However, a portion of the patients did not harbor any germline variants in the known causative genes.^13^ A recent seminal paper from Aki Kubo's group identified *FDFT1* as a novel porokeratosis gene, thereby filling a gap in the understanding of the disease mechanism.^16^ Intriguingly, some *FDFT1*‐related porokeratosis lesions had an epigenetic alteration characterized by methylation of the *FDFT1* promoter. In such cases, porokeratosis development was explained by biallelic epigenetic variants or by a combination of an epigenetic and a genetic variant. In the latter scenario, it was necessary to determine whether the somatic genetic variant was in cis or in trans relative to the epigenetic change (Figure 3a). The authors first detected adjacent heterozygous single nucleotide polymorphisms (SNPs) to determine allele configurations. Then, they elegantly combined low‐coverage long‐read sequencing targeting multiple SNPs, demonstrating that the genetic and epigenetic variants were present in trans (Figure 3b).

## 5 CLINICAL VIGNETTE 3

Recessive dystrophic epidermolysis bullosa (RDEB) is a congenital subepidermal blistering disorder characterized by skin fragility due to bi‐allelic variants in the *COL7A1* gene, which encodes type VII collagen (COL7), a major component of the anchoring fibrils present at the dermo‐epidermal junction (DEJ).^17^, ^18^ Severe RDEB manifests as extensive skin blistering and a complete absence of COL7 at the DEJ.

**FIGURE 4:**
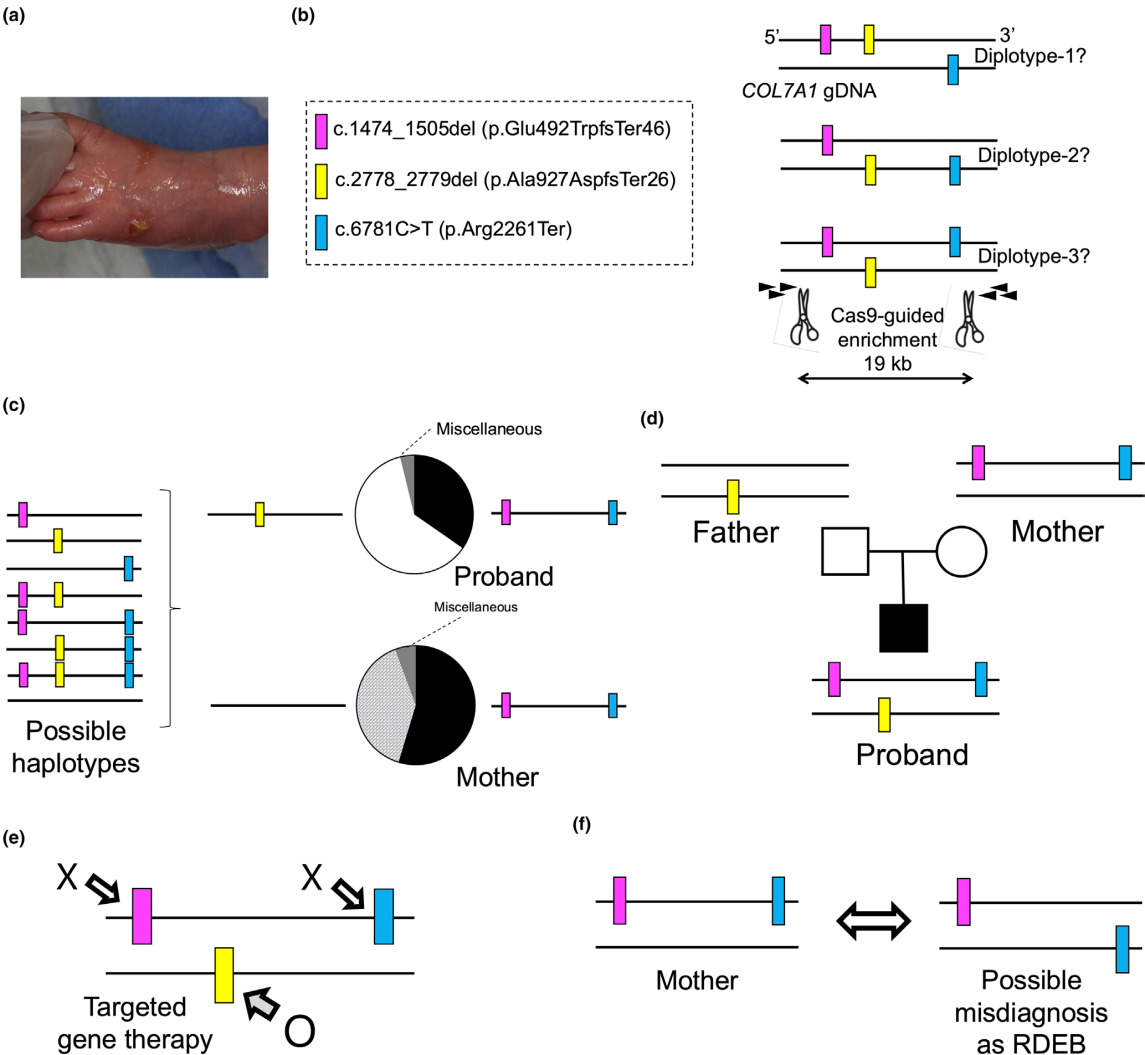
Phasing of three premature termination codon (PTC)‐causing variants in *COL7A1* using nCATS. (a) A recessive dystrophic epidermolysis bullosa (RDEB) patient who showed blisters and erosions at birth. (b) Three heterozygous PTC‐causing *COL7A1* variants were found in the patient. nCATS strategy and possible diplotypes are shown. (c) Results of nCATS from the proband and mother. (d) Phasing results from nCATS. (e) A strategy of targeted gene therapy for the proband. (f) Possible misdiagnosis of the mother as having RDEB. Images reproduced and modified with permission from Ref. [19].

My group previously encountered an RDEB case involving three heterozygous premature termination codon (PTC)‐causing variants in *COL7A1*: c.1474_1505del (p.Glu492TrpfsTer46), c.2778_2779del (p.Ala927AspfsTer26), and c.6781C>T (p.Arg2261Ter; dbSNP, rs772381373) (NM_000094.4; Figure 4a,b).^19^ The patient was born to non‐consanguineous parents. The distance between c.1474_1505del and c.6781C>T variants was 19 kb (Figure 4b). We employed nCATS and discovered that the c.2778_2779del variant was located on one allele, while the c.1474_1505del and c.6781C>T variants were present on the other allele (Figure 4c,d). Further analysis confirmed that the proband's mother was heterozygous for the allele carrying both c.1474_1505del and c.6781C>T, while the father was heterozygous for the c.2778_2779del variant^19^ (Figure 4c,d).

Phasing of these variants was crucial for designing a gene therapy approach targeting one of the three variants. In this scenario, the most effective target would be the c.2778_2779del variant (yellow), rather than c.1474_1505del (magenta) or c.6781C>T (blue) (Figure 4e) because correcting c.1474_1505del would leave the c.6781C>T variant on the same allele, and vice versa. Despite carrying two PTC‐causing variants, the mother was not affected by RDEB because both variants were located in cis, allowing the wild‐type *COL7A1* allele to produce functional COL7. Although trio analysis was possible in this scenario, nCATS was shown to be useful in phasing (Figure 4f).

## 6 CLINICAL VIGNETTE 4

Revertant mosaicism (RM) is a phenomenon in which somatic cells undergo spontaneous gene corrections in congenital diseases.^20^, ^21^, ^22^ The most likely mechanism behind RM is homologous recombination, where DNA sequences are exchanged between homologous chromosomes.^20^ In this context, RM occurs when a disease‐causing variant in an allele of somatic tissues is eliminated through homologous recombination, either via gene conversion (a non‐reciprocal exchange of DNA sequences) or intragenic crossover (a reciprocal exchange of DNA sequences). In the case of autosomal dominant diseases, the most common form of RM is loss of heterozygosity (LOH) through gene conversion, which can be detected using conventional methods like Sanger sequencing and SNP arrays to show LOH.^20^, ^23^ In pityriasis rubra pilaris type V, a rare form of autosomal dominant congenital ichthyosis, RM develops through break‐induced replication, eliminating its disease‐causing variants.^23^ In contrast, RM in autosomal recessive diseases requires more detailed investigations, particularly when intragenic crossover occurs.

Patients with epidermolysis bullosa often develop clinically revertant skin spots,^21^, ^22^ which offer a novel therapeutic opportunity in the form of epidermal autografts.^24^, ^25^ In this treatment, epidermal keratinocytes obtained from clinically revertant skin spots are cultured and the resulting cultured epidermal sheets are transplanted onto the patient's erosions or ulcers in an autologous manner. However, if the donor skin that appears clinically revertant is not truly an RM spot, the epidermal autografts may fail.^24^ The situation becomes more complicated when disease‐causing variants are missense mutations, as these variants can produce dysfunctional but residual protein expression, making RM detection through protein expression challenging.

**FIGURE 5:**
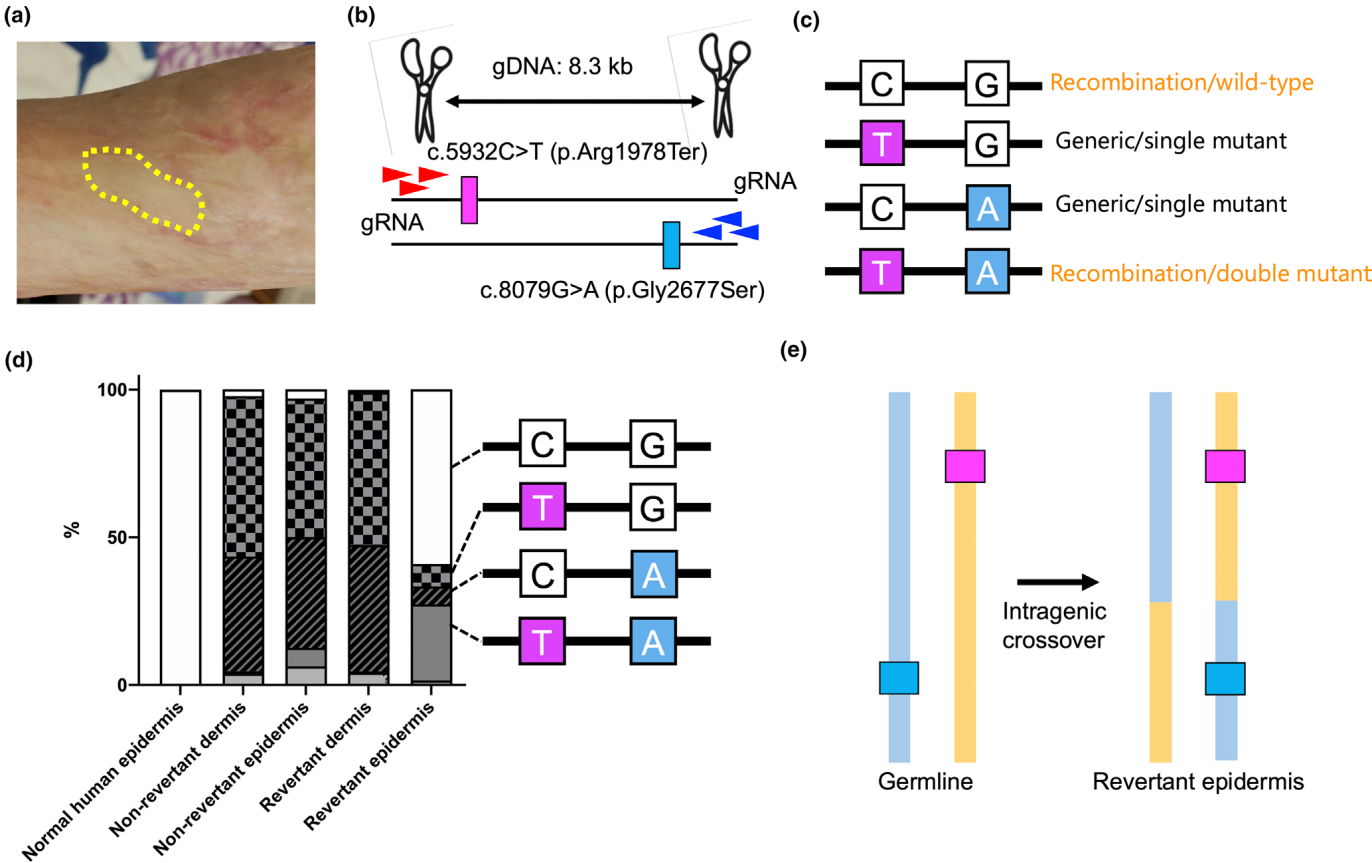
Phasing on revertant mosaicism in recessive dystrophic epidermolysis bullosa using nCATS. (a) Another RDEB patient who was compound heterozygous for c.5932C>T (p.Arg1978Ter) and c.8029G>A (p.Gly2677Ser) in *COL7A1*. A clinically revertant spot is marked with a dotted line. (b) nCATS strategy. (c) Possible haplotypes. (d) nCATS results. (e) Intragenic crossover in the revertant epidermis. Images reproduced and modified with permission from Ref. [4].

My group previously encountered an RDEB patient who was compound heterozygous for c.5932C>T (p.Arg1978Ter) and c.8029G>A (p.Gly2677Ser) in *COL7A1*, the latter being a missense variant^4^, ^26^ (Figure 5a,b). The patient developed a clinically revertant skin spot on her arm (Figure 5a), but immunofluorescence studies could not differentiate between the revertant and non‐revertant skin due to the presence of the missense variant. The distance between c.5932C>T and c.8029G>A was 8.3 kb. We performed nCATS on the clinically revertant skin and found that only the epidermis from the revertant skin exhibited RM due to intragenic crossover^4^ (Figure 5b–e).

## 7 CLINICAL VIGNETTE 5

**FIGURE 6:**
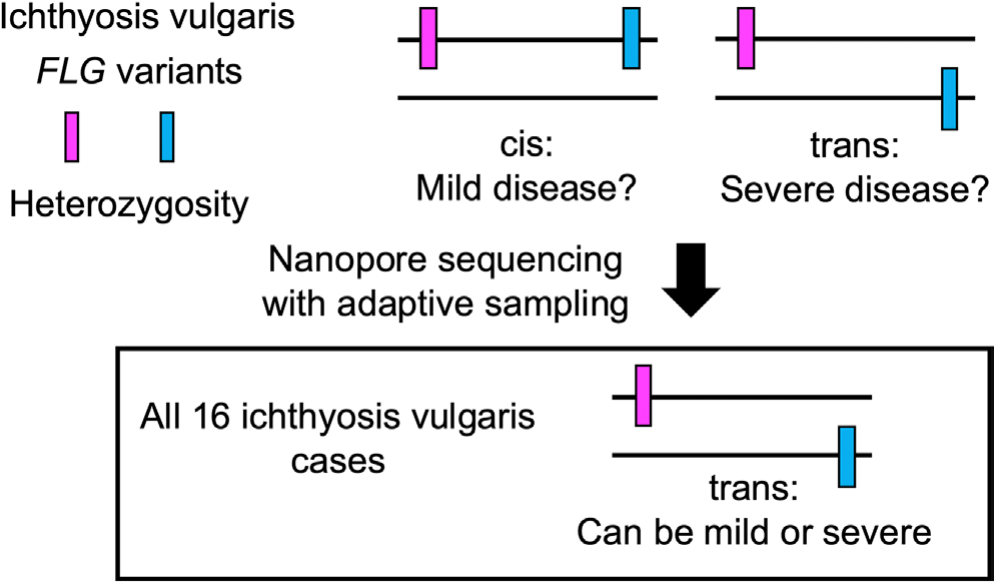
Phasing in ichthyosis vulgaris using adaptive sampling. Sixteen ichthyosis vulgaris patients with two heterozygous *FLG* variants were analyzed. Nanopore sequencing with adaptive sampling showed that all variants were present in trans for both mild and severe cases. Images reconstructed from Ref. [30].

Ichthyosis vulgaris (IV) is a common genetic disease caused by monoallelic or biallelic *FLG* variants.^27^ *FLG* variants are among the most predisposing factors for the development of atopic dermatitis.^28^, ^29^ Since the severity of IV varies significantly when two heterozygous *FLG* variants are present, it has been hypothesized that mild cases harbor the two variants in cis while severe cases are explained by the variants in trans. John Common's group tackled this problem by employing nanopore sequencing with adaptive sampling (Figure 1i) and found that all heterozygous variants in their Singaporean Chinese cohorts were present in trans, refuting the hypothesis that the cis/trans configuration of *FLG* variants affects IV severity (Figure 6).^30^ The authors further demonstrated that their strategy allowed for the simultaneous analysis of single nucleotide variants, methylation status, and copy number variants.

## 8 LIMITATIONS AND FUTURE PERSPECTIVES

As demonstrated in the clinical vignettes, phasing is essential for making accurate diagnoses and will become increasingly necessary before undertaking gene therapy. Furthermore, as illustrated in Clinical Vignette 4, accurately diagnosing RM can enable the establishment of epidermal sheets from revertant skin, which can then be used in epidermal autografts—a highly promising therapeutic approach.

As low‐coverage long‐read sequencing relies on fortuitous situations (e.g., the presence of SNPs helpful for allele configurations), enrichment of regions of interest through nCATS or adaptive sampling might be ideal for phasing. However, the efficiency of enriching the region of interest from gDNA using nCATS is extremely low, typically <1%, resulting in low coverage.^4^, ^5^, ^19^ There is also a current limitation in that the method only allows for enrichment of up to approximately 50 kb.^5^ Previously, the error rate of nanopore sequencing, which stood at a few percent, could not be entirely disregarded. However, the accuracy of nanopore sequencing has greatly improved due to advancements in nanopore devices and algorithms and now reaches over 99%.^31^, ^32^ Adaptive sampling does not need any additional experimental procedures, unlike nCATS, but it requires strong computational infrastructure. Based on the above points, nCATS would be suited for laboratories with molecular biology expertise and limited computational resources. In contrast, adaptive sampling would be ideal for laboratories with strong computational resources and bioinformatics expertise.

In the future, to enable phasing across a broader range of situations, there will be a need for the development of long‐read sequencing technologies that can produce longer, more abundant, and more accurate reads. This will also require advances in methods that preserve sufficiently long DNA fragments during extraction, the combination of nCATS or adaptive sampling with low‐cycle PCR, and the development of polymerases capable of ultralong‐range PCR.

## FUNDING INFORMATION

This work was supported by funding from the Practical Research Project for Rare/Intractable Diseases of the Japan Agency for Medical Research and Development (ID: JP24ek0109747h001).

## CONFLICT OF INTEREST STATEMENT

Ken Natsuga received grants from J‐TEC (Japan Tissue Engineering Co., Ltd.).
